# Supplementary material for: REST suppression mediates neural conversion of adult human fibroblasts via microRNA‐dependent and ‐independent pathways
Source: EMBO Mol Med. 2017 Jun 23;9(8):1117–31. doi: 10.15252/emmm.201607471 (PMC5538296; doi:10.15252/emmm.201607471)
Supplement: Supplementary file 1 — Appendix [file EMMM-9-1117-s001.pdf]

Appendix

REST suppression mediates neural conversion of adult human fibroblasts via microRNA dependent and independent pathways

Table of Contents:

Appendix Table S1. Summary of the electrophysiological properties ………………………….p.2

Appendix Table S2. Demographic information on the biopsy donors…………………………..p.3

Appendix Table S3. List of the primers used in this study……………………………...…….p.4

Appendix Table S4. Exact p-values derived from statistical tests……………………….....….p.5

Appendix Figure S1. Validation of the transgene expression in relation to its position from WPRE…………………………………………………..……………………………....p.6

Appendix Figure S2. High miRNA-9 and miRNA-124 expression following transduction with pB.mir9/124.pA. ………………………………………………………..……………..……..p.7

Appendix Table S1. Summary of the electrophysiological properties

**Intrinsic properties**	**Adult iN in vitro**	**Adult iN in vivo**
Resting membrane potential (mV)	-47.28 ± 4.05 (n=18)	-71.14 ± 3.93 (n=7)
Cell capacitance (pF)	21.78 ± 7.55 (n=22)	60.88 ± 11.99 (n=8)
Membrane resistance (MΩ)	1808 ± 308.3 (n=20)	76.63 ± 33.89 (n=8)
Number of AP able to evoke	1.35 ± 0.65 (n=20)	3.25 ± 1.35 (n=8)
N of cells with post synaptic activity	1/20	6/8

Appendix Table S2. Demographic information on the biopsy donors

**Cells source**	**Source**	**Disease**	**Sex**	**Age**	**Disease Duration (years)**	**Mutation**
Dermal biopsy	John van Geest Centre for Brain Repair	None	F	52		
Dermal biopsy	John van Geest Centre for Brain Repair	None	F	61		
Dermal biopsy	John van Geest Centre for Brain Repair	None	F	67		
Dermal biopsy	John van Geest Centre for Brain Repair	None	M	69		
Dermal biopsy	John van Geest Centre for Brain Repair	None	F	70		
Dermal biopsy	John van Geest Centre for Brain Repair	None	M	71		
Lung biopsy	Lund University, Lund	None	F	45-65		
Dermal biopsy	Lund University, Lund	None	F	74		
Dermal biopsy	Karolinska Institute, Stockholm	Genetic Alzheimer's disease	F	58	4	*APP* KM670/671NL
Dermal biopsy	John van Geest Centre for Brain Repair	Huntington's disease (41 CAG repeats)	M	61		*HTT* - 41 CAG repeats
Dermal biopsy	John van Geest Centre for Brain Repair	Sporadic Parkinson's disease	M	77	4	
Dermal biopsy	John van Geest Centre for Brain Repair	Genetic Parkinson's disease	F	55	8	*LRRK2* c.6055G>A mutation

Appendix Table S3. List of the primers used in this study

**Gene**	**Direction**	**Sequence (5’ → 3’)**
ACTB	Forward	CCTTGCACATGCCGGAG
	Reverse	GCACAGAGCCTCGCCTT
GAPDH	Forward	TTGAGGTCAATGAAGGGGTC
	Reverse	GAAGGTGAAGGTCGGAGTCA
HPRT1	Forward	ACCCTTTCCAAATCCTCAGC
	Reverse	GTTATGGCGACCCGCAG
NCAM1	Forward	GTCAGAGGCCACCGTCAACGTG
	Reverse	CTTCCCCCTCCCGGAACTCCTG
MAP2	Forward	CCGTGTGGACCATGGGGCTG
	Reverse	GTCGTCGGGGTGATGCCACG
REST	Forward	AAATGTGGCCTTAACTGGGGAA
	Reverse	TCTGTCTTTCTTCACCGACCAG
SNCA	Forward	GAGGGAGTGGTGCATGGT
	Reverse	TGCTGTCACACCCGTCAC
SYNAPSIN	Forward	CCCGTGGTTGTGAAGATGGGGC
	Reverse	TGCCACGACACTTGCGATGTCC
SYNAPTOPHYSIN	Forward	ACCTCGGGACTCAACACCTCGG
	Reverse	GAACCACAGGTTGCCGACCCAG
MAPT	Forward	CTCCAAAATCAGGGGATCGC
	Reverse	TTTTTATTTCCTCCGCCAG

Appendix Table S4. Exact p-values derived from statistical tests.

Figure	Comparison	p-values	Category	Test
Figure 1E	ABM vs. pBpA MOI 5	0.0063	**	Kruskal-Wallis test, Conover post-hoc
	ABM vs. pBpA MOI 10	0.0002	***	Kruskal-Wallis test, Conover post-hoc
	ABM vs. pBpA MOI 20	>0.0001	***	Kruskal-Wallis test, Conover post-hoc
	pBpA MOI 5 vs. pBpA MOI 10	0.0063	**	Kruskal-Wallis test, Conover post-hoc
	pBpA MOI 10 vs. pBpA MOI 20	0.0063	**	Kruskal-Wallis test, Conover post-hoc
Figure 2A	hFL1 vs. aHDF -shREST	0.0106	*	Kruskal-Wallis test, Conover post-hoc
	aHDF -shREST vs. aHDF +shREST	0.0032	**	Kruskal-Wallis test, Conover post-hoc
Figure 2D	Purity: p7 vs p10	0.0051	**	Kruskal-Wallis test; Dunn's multiple comparisons test
Figure 3F	% of TAU+ cells: CTR vs. miR-9 KD	0.0018	**	T-test
Figure EV1C	PGPA vs PBPG GFP%	0.0044		T-test
Figure EV1D	PGPA vs PBPG GFP intensity	0.0001		T-test

Appendix Figures

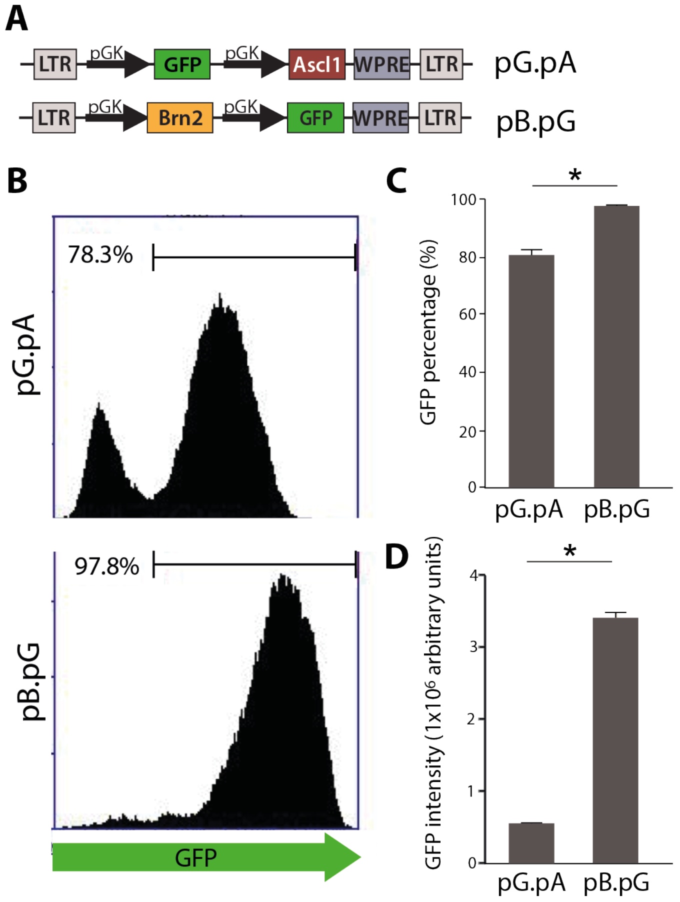

Appendix Figure S1. Validation of the transgene expression in relation to its position from WPRE. (A) Vector maps of constructs containing GFP as well as the transcription factors Ascl1 or Brn2 at different position from the woodchuck hepatitis post-transcriptional element (WPRE) at different positions. (B) Quantitative analysis of GFP showing in hFL1 transduced with the different constructs. (C) Quantifications of the percentage of cells expression GFP (D) and of the GFP fluorescence intensity in the two constructs.

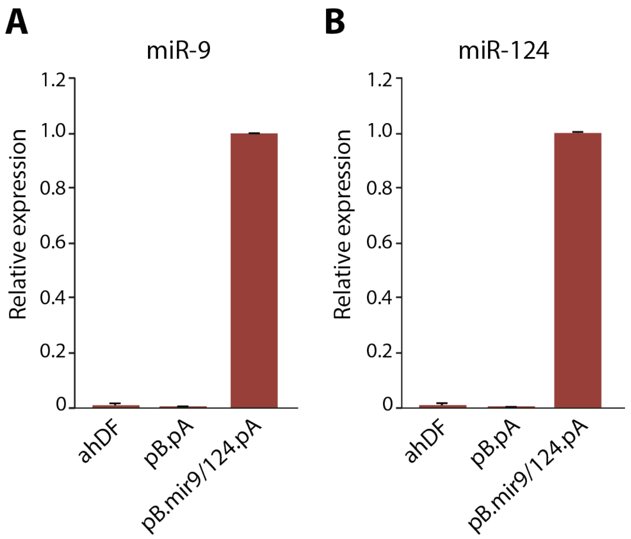

Appendix Figure S2. High miRNA-9 and miRNA-124 expression following transduction with pB.mir9/124.pA. (A, B) Quantitative PCR analysis of miR-9 (A) and miR-124 (B) three days following the transduction with either pB.pA or pB.mir9/124.pA as compared to fibroblast levels. Abbreviations: ahDF: adult human dermal fibroblasts.
